# Use of ward closure to control outbreaks among hospitalized patients in acute care settings: a systematic review

**DOI:** 10.1186/2047-2994-4-S1-O53

**Published:** 2015-06-16

**Authors:** H Wong, K Eso, A Ip, J Jones, Y Kwon, S Powelson, J de Grood, R Geransar, M Santana, AM Joffe, G Taylor, B Missaghi, C Pearce, W Ghali, J Conly

**Affiliations:** 1 W21C, University of Calgary, Calgary, Alberta, Canada; 2 University of Calgary, Calgary, Alberta, Canada; 3 AHS, Calgary, Canada; 4 University of Alberta, Edmonton, Alberta, Canada

## Introduction

Ward closure (WC) has been used as a strategy for controlling HAI outbreaks but restrict patient access, are highly disruptive and expensive.

## Objectives

We conducted a systematic review to identify studies describing the impact of WC as an intervention for outbreak containment.

## Methods

We searched MEDLINE, EMBASE, CINAHL, the Cochrane Database of Systematic Reviews, IndMED, LILACS for all years with no language restrictions plus reference lists from retrieved articles, conference proceedings and websites. Inclusion criteria were: hospitalized patients of all ages; WC as an intervention; discussion of outbreak control; and peer-reviewed. Exclusion criteria were: WC not used; full-text N/A; and studies using design or only presenting 2^0^ data analyses. Study quality was assessed with a Juni components approach. Six evaluative criteria were adapted from GRADE and the Downs and Black checklist to provide a measure for confidence in the estimate of effect of the body of evidence.

## Results

Of 97 observational studies none included a controlled comparison between WC vs other interventions. WC was used as part of a bundle of interventions precluding a determination of its direct impact either separately or in parallel, or in sequence. We also found no universal definition of WC which was widely accepted. The included studies were of poor to moderate quality; the nature of outbreak reports rendered the use of high-quality study designs and trials unfeasible.

## Conclusion

With no controlled studies identified WC remains an intervention that is not evidence-based and its use can neither be actively encouraged nor discouraged. The generalizability and applicability of WC as a control intervention could be improved by standardizing outbreak investigation reporting.

## Disclosure of interest

None declared.

